# The adapted Zelen was a feasible design to trial exercise in myeloma survivors

**DOI:** 10.1016/j.jclinepi.2020.04.004

**Published:** 2020-09

**Authors:** Joanne Land, Orla McCourt, Malgorzata Heinrich, Rebecca J. Beeken, Dimitrios A. Koutoukidis, Bruce Paton, Kwee Yong, Allan Hackshaw, Abigail Fisher

**Affiliations:** a Department of Behavioural Science and Health, University College London, WC1E 7HB, London, UK; b Research Department of Haematology, Cancer Institute, University College London, London, UK; c University College London Hospitals NHS Foundation Trust, London, UK; d Leeds Institute of Health Sciences, University of Leeds, Leeds, UK; e Nuffield Department of Primary Care Health Sciences, University of Oxford, Oxford, UK; f NIHR Biomedical Research Centre, Oxford University Hospitals NHS Foundation Trust, Oxford, UK; g Institute of Sport Exercise & Health, London, UK; h Cancer Research UK & UCL Cancer Trials Centre, University College London, London, UK

**Keywords:** Research design, Exercise-oncology, Exercise, Physical activity, Multiple myeloma, Post randomization consent

## Abstract

**Objectives:**

We used a method rarely seen in cancer behavioral trials to explore methods of overcoming difficulties often seen in randomized controlled trials. We report our experiences of the adapted Zelen design, so that other researchers can consider this approach for behavioral trials.

**Study Design and Setting:**

The adapted Zelen design was used to explore the effects of exercise on multiple myeloma patients fatigue, quality of life, and physical outcomes. All participants consented to an observational cohort study of lifestyle factors but were unaware of subsequent randomization to remain in cohort only group or be offered an exercise intervention requiring second consent.

**Results:**

There was lower than expected uptake to the exercise offered group (57%), so the length of recruitment increased from 24 to 29 months to ensure power was reached. At enrollment, patients were unaware of the potential increased commitment, and as a result, 62% of participants allocated to the intervention declined because of the extra time/travel commitment required. This emulates clinical settings and suggests improvements in intervention delivery are required. Our findings suggest that the adapted Zelen design may be useful in limiting dropout of controls due to dissatisfaction from group allocation, or contamination of control arm.

**Conclusion:**

Future use of this design warrants careful consideration of the study resources and recruitment time frames required but holds potential value in reducing contamination, control group dissatisfaction, and resulting dropout. Adapted Zelen design reduces selection bias and therefore gives clinicians a better understanding of acceptability in clinical settings. Future studies should evaluate control group experiences of the design and formally record contamination throughout the study to confirm its acceptability.

**What is new?**

**Key findings**
• The adapted Zelen design reduces selection bias and can provide real-world acceptability of an exercise intervention within a clinical service.
• Dropout rates are comparable with a cross-over design without the associated costs and time.
• An advantage of the adapted Zelen design is that no patients were lost from usual care because of dissatisfaction of allocation.

**What this adds to what was known?**
• The adapted Zelen design increases demand on research resources, as a larger number of participants are required to achieve power than a randomized controlled trial. However, it provides an acceptable alternative study design to avoid common disadvantages found with other study designs, for example, dropouts because of dissatisfaction at allocation, contamination, and low generalizability in clinical settings.

**What is the implication and what should change now?**
• To date, there has been no qualitative work in adapted Zelen trials for cancer patients to explore their experiences of being involved in these study designs. This study gathered brief insights from intervention group participants, but its imperative further work is done to gather greater understanding of its strengths and applicability. This would aid the ethical debate surrounding this design.
• Further work is required to eliminate barriers preventing patients from accessing exercise interventions and find acceptable alternatives to face-to-face groups.

## 1 Background

There is growing interest in using alternative study designs to address pitfalls associated with traditional designs. In this article, we describe our experience of using an “adapted Zelen” design within an exercise oncology setting in patients with a hematological cancer: multiple myeloma (MM).

The randomized controlled trial (RCT) has long been accepted as the “gold standard” for testing clinical interventions. Pragmatic RCTs (which aim to inform routine health decision-making) compare an intervention to usual care/standard care. All patients undergo “fully” informed consent—that is to say—before randomization to groups, all potential trial participants are informed that they may be randomly selected to the intervention group and offered a nonstandard (new/experimental) treatment or usual care/standard care. This can pose significant challenges because it is not possible to blind participants to their group allocation in behavioral studies [1]. Participants who take part in standard RCTs will make a judgment of their preferred treatment and often expect/hope to be allocated to the treatment group [2]. If this does not occur, it can be followed by dissatisfaction, discontent with the research process, and distrust in those who approached them to take part [3], particularly when target patients are highly motivated to engage in self-management strategies [4]. In particular, cancer patients are becoming increasingly aware of the potential benefits of exercise, especially in relation to recovery from their treatment [5] and is a key motivator for them to enroll in RCTs of exercise interventions [6].

Consequently, randomization to a “no exercise” or control group may lead to dropout after allocation or self-initiation of exercise, having been alerted to it in the trial information sheet [6]. This creates a bias that dilutes the true intervention effect (contamination of the control group), which is likely to be a major contributing factor to the often small to moderate effect sizes observed in cancer exercise RCTs [7]. For example, an exercise study among colorectal cancer survivors found no significant differences between the control and exercise intervention group and attributed this to the high contamination rate (51% of the control group engaged in >60 minutes of moderate to strenuous exercise) [4].

Several methods have been used in cancer exercise trials to attempt to minimize contamination and dropout in control groups. A systematic review of 40 studies addressing this concluded that a trial design that offered the control group an alternative intervention during the study or offered the intervention to them at a later stage in a cross-over or wait list control design could be an effective way of minimizing contamination and dropout [7]. However, these designs are time consuming, expensive, and limit the possibility of testing intervention effects long-term, as they are no longer “true” controls.

## 2 Zelen design

One way of eliminating the aforementioned challenges is through the use of the Zelen design. The original Zelen design involved randomization before consent, with consent only required from those allocated to the intervention, whereas the control group receive their usual care [8]. One of the main features of the original Zelen design is that informed consent is not required from the control group. Baseline characteristics and outcomes are collected from medical records (with ethical approval). However, it is not possible to have interaction with the control group during follow-up, as they are not informed of their presence in a study, and this can reduce possible bias introduced by the nature of study participation and undergoing additional assessments but could be considered unethical [9]. To overcome this issue, an alternative Zelen design, which uses a double consent process, an “adapted Zelen design” was used (described below) [8,10].

The adapted Zelen has two stages of consent. In the first stage, informed consent is sought from all participants to a cohort lifestyle study. They are then randomized without knowledge, and in the second stage, only participants who have been assigned to the intervention are reapproached and given information about it. At this stage, if they agree to participate, they are asked to give second informed consent for the intervention, those who decline remain in the cohort study. Those in the cohort only group are not informed about the randomization or the intervention trial. The design has made an important contribution to evidence-based medicine [10]. However, it has rarely been used in exercise trials and has mixed support [6,10,11]. Campbell et al. conducted a Zelen study of a physiotherapy intervention nested within an observational study for patients with chronic arthritis. They found it an acceptable method, with a 64% participation rate, which they reported as higher than conventional RCTs in the field. They successfully avoided contamination in the control group and had negligible loss to follow-up [11]. We have only found one cancer exercise trial using the Zelen design. Velthuis et al. investigated the effect of physical activity (PA) on cancer-related fatigue and quality of life (QoL) on 64 breast and colon cancer patients [6]. However, because of the ethics committee deeming it not ethical that participants are not aware of the randomization process, they had to modify the design and consent patients to baseline assessments in addition to postponement of information about the study at the end. They concluded that the Zelen design with consent to postponed information at the end of the study was not any better than conventional randomization because of a high dropout from the intervention and low overall participation in the study, which they attributed to postponed information [6,12]. These mixed findings highlight the need to evaluate the double-consent Zelen design in other oncology exercise trials. This report describes these challenges and our experience of trying to overcome them using an adapted Zelen design in a trial of an exercise intervention in patients with MM.

## 3 Development of “Myeloma Advancing Survivorship Cancer Outcomes Trial”

There is strong evidence that exercise improves outcomes after a cancer diagnosis in patients with breast, prostate, and colorectal cancers [13,14]. However, it is unclear if exercise is also beneficial in hematological cancers, such as MM. We previously undertook a pilot study in 37 patients, which suggested that exercise intervention improved QoL, fatigue, and upper and lower limb strength, these preliminary data require confirmation with a larger RCT [15]. Therefore, we conducted the Myeloma Advancing Survivorship Cancer Outcomes Trial (MASCOT; ISRCTN 38480455), which tested the hypothesis that a physiotherapist-led individually tailored exercise program would improve symptoms of cancer-related fatigue when compared with usual care, as well as explore its effect on several clinical, physical, and psychosocial outcomes [16].

However, the challenges of traditional RCTs were likely to be particularly pertinent in MM, an incurable cancer that is associated with bone destruction, pain, fractures, and deconditioning [17]. For most patients, response to initial combination chemotherapy can be followed by a prolonged period (median 2–3 years) where disease is stable. Patients are generally highly motivated to undertake strategies that can help them recover from the effect of treatment [18]. Considering a pilot study had been conducted at the center previously, we believed our patients may have some awareness of the benefits observed, and we, therefore, believed that randomization to a no exercise arm would result in patient distress and consequently lead to high dropout rates and contamination [19]. A cross-over or wait list control was deemed not feasible because of the time, cost, and desire to follow patients up longer term.

**Fig. 1:**
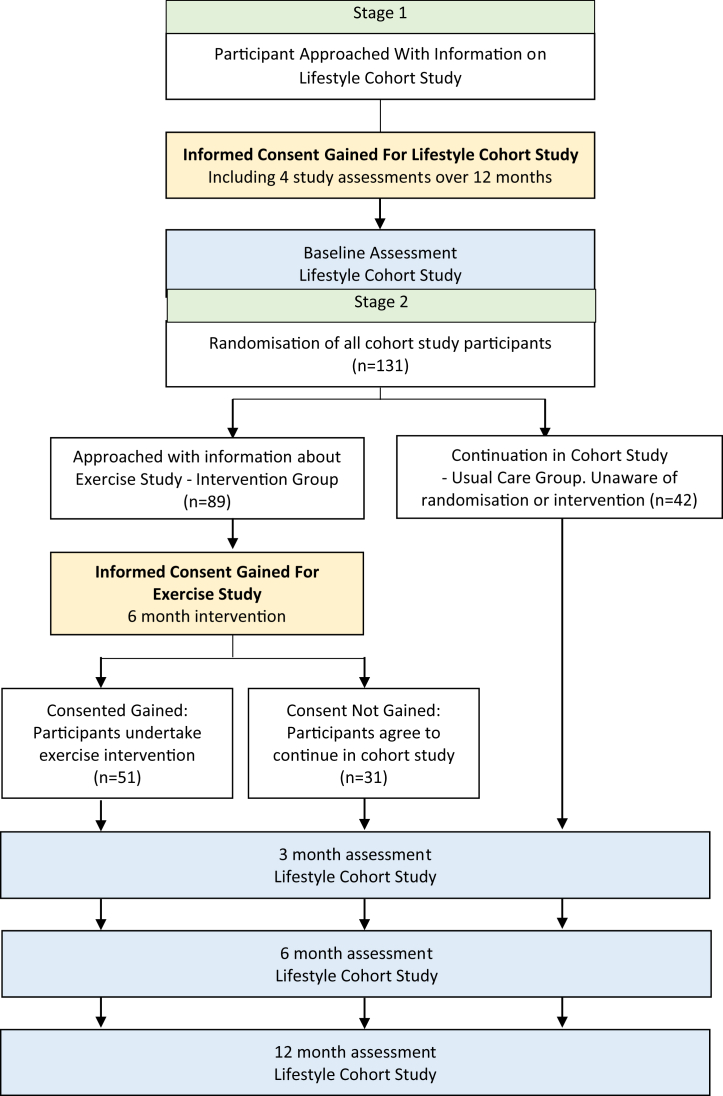
Flow of participants through the double consent process.

To address these issues, MASCOT used an adapted Zelen design of an exercise RCT embedded within a longitudinal lifestyle cohort study [16]. Patients were identified and screened by clinicians in MM clinics to assess eligibility, and those eligible were approached by the research team. MASCOT had two stages of consent. In stage 1, patients were sent an information sheet inviting them to participate in a “Lifestyle Cohort Study” aimed at increasing understanding of the relationship between lifestyle behaviors and symptoms such as fatigue and QoL and how these changed over time. The details of the lifestyle cohort study are published elsewhere [16]. Importantly, this information did not mention PA and its potential benefits for MM patients as a focus of the research. After consent was obtained to the Lifestyle Cohort Study, baseline assessments were completed. Participants were then randomized, without their knowledge, to either be offered the exercise intervention (“exercise offered”) or not (“cohort only”). In stage 2, those who were randomized into the exercise offered group were approached by a researcher and invited to take part in a “second” study evaluating an exercise intervention. Fig. 1 illustrates the flow of participants through the double consent process. Those in the cohort only group were not informed about the randomization process or existence of the exercise intervention. The measurement schedule (0, 3, 6, and 12 months) and outcome measures were the same for both groups. Only the exercise intervention was part of the second consent. The intervention was delivered by a physiotherapist in the hospital gym. During the first 3 months, patients attended the gym once per week and were instructed to exercise at home twice a week. During the following 3 months, the intensity of the intervention was reduced to one monthly gym session and three home sessions per week. During the last 6 months, participants were only instructed to exercise at home three times per week.

Full ethical approval for this methodology was provided by Queens Square Ethics Committee (13/LO/1105). Five researchers of the MASCOT team attended the ethics meeting, including a myeloma consultant (KY), behavioral scientists (AF and RB), a senior physiotherapist (BP), and the trial statistician (AH). This design can evoke strong emotions and rejection from ethicists and researchers [20], and we believe that the attendance of our diverse team providing the rationale behind the design helped mitigate a lengthy review.

### 3.1 Recruitment

Overall, 313 patients were invited by post to take part in the lifestyle cohort study. Of these, 80 (26%) declined, 64 (20%) never responded, 23 (7%) were medically excluded, and 8 (3%) became ineligible. Between June 2014 and November 2016, 138 patients (44%) consented to take part. After consenting, one patient (0.3%) withdrew and six patients (2%) relapsed. Therefore, 131 patients were enrolled in the lifestyle cohort study and were eligible for randomization (Fig. 1). Initially, randomization was proposed at a 1:1 ratio. As the study progressed, the number of participants declining the intervention after randomization was 38%, which was greater than we expected, given our pilot [15], which had a 20% decline rate. Thus, the randomization allocation ratio was altered to 3:1 in favor of the intervention to ensure that the study maintained statistical power (34 patients were required in the exercise group to detect an effect size of 0.69, as per the power calculation).

Of the 131 participants who were randomized, 89 were offered the exercise intervention. Of these, 34 participants (38%) declined and four (6%) became ineligible because of disease relapse, resulting in an uptake of 51 (57%). The main reasons for declining the intervention were time/travel commitment (62%, *n* = 21). Additional reasons were patients perceiving medical problems as a barrier (12%, *n* = 4), nonresponse to invitation (9%, *n* = 3), on long-term holiday (9%, *n* = 3), other reasons (5%, *n* = 2), and withdrew (3%, *n* = 1).

At 3 months, one participant (2%) withdrew from the cohort only group citing personal family issues as the reason, and 8 participants (19%) withdrew from the “exercise offered” group (five of whom consent to exercise and began the intervention and three who had never consented to exercise). These dropout rates are comparable to other oncology exercise studies [7] and suggest we did not lose participants from the cohort only group because of dissatisfaction of allocation. There were no differences between the characteristics of those who agreed and declined to take part in the intervention.

## 4 Discussion

A double-consent adapted Zelen design method was used over a traditional RCT to reduce patient distress, contamination, and dropout in the cohort only group, allowing us to explore whether exercise is a safe and effective treatment for MM survivors. Several important learning points around recruitment, randomization, and contamination were noted, which may aid future study design in this area.

Using the adapted Zelen design, we aimed to overcome the selection bias so often reported in exercise trials (i.e., trials tend to enroll self-selected participants who are motivated to exercise) [4]. However, recruitment to the lifestyle cohort study had a lower uptake (44%) than expected, and based on the rate of 80% in the earlier single-arm pilot exercise trial, we anticipated more patients would take up the intervention [15]. Possibly potential participants for the lifestyle cohort study may have perceived little benefit from enrolling in the study. In addition, only 57% then agreed to participate in the exercise intervention arm (although this is in line with a systematic review of 65 cancer exercise trials using a standard RCT design where uptake was estimated at 63% (range 33–80%) [21].

As a specialist center, many of our participants had to travel considerable distances to attend the intervention. Despite reimbursement for travel, 62% of those who declined the exercise intervention felt the increased time and number of visits were a barrier to taking part as they were not expecting this increase in commitment. These reasons are similar to those cited in other cancer exercise trials [3]. Velthuis et al. who used the Zelen design reported a low inclusion rate of 40% of eligible patients. They attributed it to similar reasons to those of the MASCOT study, with their participants also citing lack of information regarding the increased efforts expected of them [6].

Although it could be argued that the rate of declining to take part in the intervention in our study was fairly high, this may suggest lack of awareness of the randomization and low expectations of engagement were unforeseen barriers to recruitment. The Zelen design enables researchers to see the acceptability of the intervention in real-world settings by reducing selection bias. Our results suggest that our intervention was within similar uptake ranges to other exercise trials, and the reasons provided for declining the intervention in our study were similar to a standard RCT design [22]. It should be highlighted that is may not be an issue with the Zelen design, but could also be that inverventions are not accessible or attractive to patients, and this needs to be addressed.

During the trial, we realized that the target sample size would not be reached because of the low uptake of the intervention. Therefore, we changed the randomization process to have unequal allocation, which Avins argues the loss of power or total numbers of required subject is small with ratios of 2:1 and only slightly more for 3:1 [23]. The allocation ratio was specified as 3:1, favoring the exercise intervention, based on the observed uptake rate, thereby allowing us to complete recruitment. We anticipated 24 months to recruit, which ended up being 30 months. Researchers should be made aware that using an adapted Zelen may require a longer than expected recruitment period and should be cautious when estimating uptake.

The risk of contamination in control groups has been reported in exercise trials. Persoon et al. investigated the effect of a supervised exercise intervention on physical fitness and fatigue in myeloma and lymphoma patients after autologous stem cell transplant. There were no significant differences reported between groups in a range of outcomes, and the authors described likely contamination with 47% of the control group reporting participation in 10 or more sessions of physiotherapy during the trial period. This was attributed to a recent increase in cancer patients’ awareness of cancer rehabilitation in the Netherlands [24].

An advantage of the adapted Zelen is to reduce contamination. However, this is compromised if the control group becomes aware of the intervention, and therefore, the same threats as seen within RCTs of dropout or initiation of exercise can still occur [25]. We had anecdotal reports of contamination from the cohort only group who reported discussing MASCOT with one another at weekly myeloma outpatient clinics and support groups that are run at our center. Consequently, participants may have been aware of the exercise study before consenting to the lifestyle cohort. This was captured within the qualitative studies we undertook as part of the main study (Box 1).

Box 1 **Qualitative interview quotes from Myeloma Advancing Survivorship Cancer Outcomes Trial participants.**

**Table:** 

Themes	Quotes illustrating
Participant discussing exercise study with others	Q: Would you recommend taking part in the study to other people? A: “Yeah. Yeah. I have done.” Q: You have done? Is that other people that you've met? A: “Through the clinic, yeah, through the hospital here.”
Participant awareness of the exercise intervention	Q: When they introduced the exercise element, were you surprised? A: “You, you mean the every week one? Um, yeah, I guess to a certain extent I was because [the doctor] had not suggested that [the exercise aspect of the study] at the outset and then [the researcher] suggested that it was, you know, secret, almost …um, which I found quite perverse but there we are. Um, but it took on a different dimension.”

Velthuis et al. reported similar problems with patients meeting in chemotherapy day care and discussing the study, whereas Campbell et al. did not report any contamination [6,11]. In the latter study, patients with arthritis were contacted by phone in the community, which meant they did not meet other participants, thus reducing the opportunity to be made aware of the full study [11]. However, our results do not suggest that the cohort only group were meaningfully affected by contamination, as there were significant improvements in leg strength in favor of the intervention group and PA levels in cohort only group reduced at each time point (data to be published). There is no formal documentation from other Zelen studies about the level of contamination in the control group to assess potential dilution of effect size. We did not document evidence for contamination in this study, but this is warranted in future studies using similar design.

With a traditional RCT design, participants may drop out because of disappointment at their allocation, and a systematic review into control group dropout rates in cancer exercise trials showed that the largest mean percentage dropout was found in studies where no intervention was provided to the control group (11.2%, SD 8.1) [7]. Although we did not provide any treatment to the cohort only group, our dropout rate at 3 months was 5%, which is comparable with the study design that reports the lowest dropouts by providing some form of an intervention to the control group after the study (5.8%, SD 5.0) [7]. This may be a promising indication that the adapted Zelen could potentially reduce dropout rates because of treatment allocation without providing the intervention.

To the best of our knowledge, there are no studies reporting cancer patients' thoughts about the Zelen design. Qualitative data from participants in a neonatal clinic elicited mixed attitudes toward Zelen randomization, with participants evenly split between accepting and not accepting the design. Some reported that they found the Zelen design underhand yet acknowledged if you were in the control group, it was kinder than an RCT, as you were not aware you had not received a potentially desirable treatment [26].

We used this design over a standard RCT because we felt it would cause patient distress if they were not allocated to the exercise intervention and may lead to contamination and participants to drop out. However, it is worth acknowledging that historically, this design has previously led to ethical criticism because although it spares emotional distress for the participant, it raises questions as to whether it is ethical for patients to be randomized without knowledge or consent [19].

Reporting an alternative study design similar to Zelen, Gal et al. used a “trials within cohort RCT” within an exercise oncology setting [27]. In the first stage, participants enroll in a cohort study and provide broad consent to be approached for any experimental interventions or to be a control for multiple studies that may be undertaken during the cohort study period. In the second stage, informed consent is only sought in those randomly allocated to the intervention. In the third stage, all cohort participants are informed of the multiple RCT results. Kim et al. reported it is ethically superior to the adapted Zelen design because patients are fully informed they will be randomized [28]. Gal et al. reported no contamination, and this design also mitigates patient distress because they remain unaware of the interventions unless they are selected [27]. This design is suggested to overcome the ethical disadvantages of the Zelen design and provides advantages over traditional study designs, and researchers may want to explore this as an alternative to the Zelen design.

It is important to note that in the adapted Zelen design, the control patients are not deprived of any other treatments and are managed as per usual clinical practice. Behavioral interventions are generally expected to only lead to small to moderate benefits. Therefore, the negative impact of contamination can be substantial, and this can only be mitigated by a Zelen type of design. We only interviewed patients who took up the exercise intervention, but although most participants were not aware that they had been randomized as a result of consenting to the lifestyle study, one participant did report that they thought it was perverse (Box 1). It is possible that in those motivated to exercise in our study, we may have reduced potential distress for the patient at the time of enrollment, but it remains unclear whether it caused delayed distress once those who were randomized to the cohort only group realized they were not fully informed. We would recommend researchers considering using the Zelen design collect data on control participants’ experience to assess its acceptability within cancer trials and aid the ethical debate.

## 5 Conclusion

The adapted Zelen presents an alternative way to reduce the risk of contamination and bias in exercise oncology research where blinding participants to their allocation and the intervention they receive is difficult. By reducing selection bias, it provides real-world acceptability of an intervention.

Further work is warranted to explore the experiences of participants in both arms to gather a greater understanding of its strengths and applicability.

## CRediT authorship contribution statement

**Joanne Land:** Investigation, Data curation, Writing - original draft. **Orla McCourt:** Investigation, Data curation, Writing - original draft. **Malgorzata Heinrich:** Investigation, Data curation. **Rebecca J. Beeken:** Conceptualization, Methodology, Writing - original draft. **Dimitrios A. Koutoukidis:** Investigation, Data curation, Formal analysis, Writing - original draft. **Bruce Paton:** Conceptualization, Methodology, Investigation, Data curation. **Kwee Yong:** Conceptualization, Methodology, Formal analysis, Funding acquisition, Project administration, Supervision, Writing - original draft. **Allan Hackshaw:** Conceptualization, Methodology, Formal analysis, Writing - original draft. **Abigail Fisher:** Conceptualization, Methodology, Formal analysis, Funding acquisition, Project administration, Supervision, Writing - original draft.

## Supplementary data

Data Profile
